# HIV-1 Drug-Resistance Surveillance among Treatment-Experienced and -Naïve Patients after the Implementation of Antiretroviral Therapy in Ghana

**DOI:** 10.1371/journal.pone.0071972

**Published:** 2013-08-19

**Authors:** Nicholas I. Nii-Trebi, Shiro Ibe, Jacob S. Barnor, Koichi Ishikawa, James A. M. Brandful, Sampson B. Ofori, Shoji Yamaoka, William K. Ampofo, Wataru Sugiura

**Affiliations:** 1 Department of Medical Laboratory Sciences, School of Allied Health Sciences, University of Ghana, Accra, Ghana; 2 Department of Infection and Immunology, Clinical Research Center, National Hospital Organization Nagoya Medical Center, Nagoya, Japan; 3 Department of Virology, Noguchi Memorial Institute for Medical Research, University of Ghana, Accra, Ghana; 4 AIDS Research Center, National Institute of Infectious Diseases, Tokyo, Japan; 5 Department of Medicine, Koforidua Regional Hospital, Koforidua, Ghana; 6 Department of Molecular Virology, Graduate School of Medicine, Tokyo Medical and Dental University, Tokyo, Japan; 7 Department of AIDS Research, Nagoya University Graduate School of Medicine, Nagoya, Japan; University of Hawaii Manoa, United States of America

## Abstract

**Background:**

Limited HIV-1 drug-resistance surveillance has been carried out in Ghana since the implementation of antiretroviral therapy (ART). This study sought to provide data on the profile of HIV-1 drug resistance in ART-experienced and newly diagnosed individuals in Ghana.

**Methods:**

Samples were collected from 101 HIV-1-infected patients (32 ART-experienced cases with virological failure and 69 newly diagnosed ART-naïve cases, including 11 children), in Koforidua, Eastern region of Ghana, from February 2009 to January 2010. The *pol* gene sequences were analyzed by in-house HIV-1 drug-resistance testing.

**Results:**

The most prevalent HIV-1 subtype was CRF02_AG (66.3%, 67/101) followed by unique recombinant forms (25.7%, 26/101). Among 31 ART-experienced adults, 22 (71.0%) possessed at least one drug-resistance mutation, and 14 (45.2%) had two-class-resistance to nucleoside and non-nucleoside reverse-transcriptase inhibitors used in their first ART regimen. Importantly, the number of accumulated mutations clearly correlated with the duration of ART. The most prevalent mutation was lamivudine-resistance M184V (*n* = 12, 38.7%) followed by efavirenz/nevirapine-resistance K103N (*n* = 9, 29.0%), and zidovudine/stavudine-resistance T215Y/F (*n* = 6, 19.4%). Within the viral protease, the major nelfinavir-resistance mutation L90M was found in one case. No transmitted HIV-1 drug-resistance mutation was found in 59 ART-naïve adults, but K103N and G190S mutations were observed in one ART-naïve child.

**Conclusions:**

Despite expanding accessibility to ART in Eastern Ghana, the prevalence of transmitted HIV-1 drug resistance presently appears to be low. As ART provision with limited options is scaled up nationwide in Ghana, careful monitoring of transmitted HIV-1 drug resistance is necessary.

## Introduction

The number of people worldwide living with HIV/AIDS in 2010, according to the latest report from the United Nations Programme on HIV/AIDS, was estimated to be 34.0 million [1]. Although the highest prevalence of HIV/AIDS remains in sub-Saharan Africa, current massive and rapid scaling up of antiretroviral therapy (ART) has resulted in the decline of the epidemic in this region [1]. Indeed, HIV prevalence in Ghana gradually declined from a peak of 3.6% in 2003 to 2.1% in 2011 due to the National AIDS Control Programme implementing a strategy for achieving universal access to ART. The program has been continuously expanding since 2003, and the coverage of ART in 2011 was estimated to be 26.6% (59,007/221,884) and 63.6% (8,057/12,661) for total HIV-infected individuals and for HIV-positive pregnant women, respectively [2].

The first-line regimen of ART recommended in Ghana is the combination of two nucleoside reverse-transcriptase inhibitors (NRTIs) and a non-nucleoside reverse-transcriptase inhibitor (NNRTI) [3]. Specifically, the two NRTIs selected are lamivudine (3TC) and either zidovudine (AZT) or stavudine (d4T), then either nevirapine (NVP) or efavirenz (EFV) as the NNRTI [3]. For the second-line regimen in Ghana, two protease inhibitors (PIs) are available, nelfinavir (NFV) or lopinavir/ritonavir (LPV/r), either of which is recommended to use with two NRTIs, abacavir (ABC) and either tenofovir (TDF) or didanosine (ddI) [3].

Drug-resistant HIV variants selected during ART have the potential to be transmitted to others. Indeed, drug-resistant HIV has been widely described in ART-naïve individuals. For example, a recent systematic review revealed that the overall prevalence of drug-resistant HIV-1 transmission reached 12.9% in North America, 10.9% in Europe, 6.3% in Latin America, 4.7% in Africa, and 4.2% in Asia [4]. Thus, the higher prevalence of drug-resistant HIV-1 transmission has been reported in higher ART coverage areas, mostly in developed countries. It is important to note that, along with ART scale-up, the prevalence of transmitted HIV-1 drug-resistance increased from 2.8% before 2001 to 5.3% after 2003 in African countries [4]. As the transmission of drug-resistant HIV may seriously affect the efficacy of first-line ART, surveillance to monitor the prevalence of transmitted HIV drug-resistance has become an important issue in African countries. The prevalence of transmitted HIV-1 drug resistance in Ghana was reported in two studies. One was conducted in 2003 in the Greater-Accra Region of Ghana [5], and the other one was conducted between 2002 and 2004 in Accra and two sites of the Eastern region, Agomanya and Atua [6]. Both studies reported no case of drug-resistant HIV-1 transmission [5], [6]. As at December 2009, the national response had established programs for the provision of ART in hospitals and health centers in several districts in the ten regions of Ghana [7]. However, since ART was expanded in Ghana, the situation of transmitted HIV-1 drug-resistance has not been reported.

To clarify the prevalence, pattern, and spectrum of HIV-1 drug resistance in the era of scaled up ART in Ghana, particularly in ART-experienced patients and transmission to new individuals, we surveyed HIV-1 drug resistance among ART-experienced and -naïve patients enrolled between 2009 and 2010 in Koforidua, the capital of the Eastern region, Ghana. Concomitantly, we analyzed HIV-1 subtypes in detail to further understand the epidemiology of HIV-1 infections in Ghana.

## Methods

### Patients

HIV-infected patients who visited the Koforidua Regional Hospital (KRH) from February 2009 to January 2010 were enrolled in the study. KRH is the main HIV/AIDS clinic in the capital of the Eastern region of Ghana. This hospital is responsible for HIV prevention and intervention programs in the area and provides free ART with care and support to HIV-infected patients. The Institutional Review Board of the Noguchi Memorial Institute for Medical Research granted ethical approval for this study. All patients or their caregivers gave written consent to participate in the study.

### CD4^+^ T-cell Count and Plasma HIV-1 Viral Load Monitoring

For an indication of immune status, CD4^+^ T-cells were measured using a FACSCount flow cytometer (Becton Dickinson, San Jose, California, USA). Plasma HIV-1 viral loads (pVLs) were quantified using an in-house real-time reverse-transcription and polymerase chain reaction (RT-PCR) assay as previously reported [8]. ART-experienced patients with pVL>150 copies/mL were considered as virological failures.

### HIV-1 Drug-resistance Genotyping

HIV-1 drug-resistance genotyping was performed as previously reported with some modifications [9]. In brief, viral RNA was extracted from 200 µL of plasma samples using QIAamp viral RNA mini kit (Qiagen, Hilden, Germany). RT-PCR was performed with QIAGEN one-step RT-PCR kit (Qiagen), and nested PCR was subsequently performed using AmpliTaq DNA polymerase (Applied Biosystems, Foster City, USA). Specific primers known as DRPRO5, DRPRO2L, DRPRO1M, and DRPRO6 were used for the protease (PR) region (424 bp, positions 2,168 to 2,591 in the reference HXB2 sequence), and DRRT1L, DRRT4L, DRRT7L, and DRRT6L primers for the reverse transcriptase (RT) region (838 bp, positions 2,510 to 3,347) [9]. Details of the primers used in the study are shown in Table 1. Nucleotide sequencing was performed using ABI 3730 auto-sequencer followed by editing with SeqScape software v2.5 (Applied Biosystems). HIV-1 drug-resistance mutations were detected according to the latest definition of the International AIDS Society-USA panel [10]. In addition, transmitted HIV-1 drug-resistance mutations were defined using the mutation list proposed by Bennett et al. [11].

**Table 1:** List of primers used in HIV-1 genotypic drug-resistance testing.

Target region	Amplicon		Primers			
	Size (bp)	Position^a^	Reaction	Direction	Name	Nucleotide sequence (5′ to 3′)
Protease	424	2,168 to 2,591	RT-PCR	Forward	DRPRO5	AGA CAG GYT AAT TTT TTA GGG A
				Reverse	DRPRO2L	TAT GGA TTT TCA GGC CCA ATT TTT GA
			Nested PCR	Forward	DRPRO1M	AGA GCC AAC AGC CCC ACC AG
				Reverse	DRPRO6	ACT TTT GGG CCA TCC ATT CC
Reverse transcriptase	838	2,510 to 3,347	RT-PCR	Forward	DRRT1L	ATG ATA GGG GGA ATT GGA GGT TT
				Reverse	DRRT4L	TAC TTC TGT TAG TGC TTT GGT TCC
			Nested PCR	Forward	DRRT7L	GAC CTA CAC CTG TCA ACA TAA TTG G
				Reverse	DRRT6L	TAA TCC CTG CAT AAA TCT GAC TTG C

bp, base pairs; PCR, polymerase chain reaction; and RT-PCR, reverse transcription and polymerase chain reaction.

a Amplicon positions in the reference HIV-1 HXB2 sequence are represented.

### HIV-1 Subtyping

HIV-1 subtyping was performed using the *pol* gene sequences (1,095 bp, positions 2253 to 3347). Phylogenetic tree was constructed with the references of subtypes A-D, F-H, J, K, and all circulating recombinant forms (CRFs) 01 to 51, except 30, 41, and 50, obtained from the HIV Sequence Database at the Los Alamos National Laboratory. In addition, HIV-1 sub-subtype A3 (DDI579, DDJ360, and DDJ369) and A4 (97CD_KCC2, 97CD_KTB13, and 02CD_KTB035) isolates were added to the phylogenetic tree analysis, as these sub-subtypes have been reported as circulating in several African countries [12], [13]. Multiple sequences were aligned using the MUSCLE program, and genetic distances were calculated based on the maximum composite likelihood model using MEGA software v5.05 [14]. Phylogenetic trees were constructed using the neighbor-joining method with 1,000 bootstrap replicates. In similarity plotting and boot-scanning analyses, nine HIV-1 subtypes, A-D, F-H, J, and K, and three CRFs, CRF02_AG, CRF06_cpx, and CRF09_cpx, were used as references. Similarity plotting and boot-scanning were performed using SimPlot software v3.5.1 with window and step sizes of 250 and 20 nucleotides, respectively [15]. One HIV-1 isolate identified with an unknown mosaic pattern both in similarity plotting and boot-scanning analyses was considered as a unique recombinant form (URF).

### Statistical Analysis

The Fisher’s exact test and the Mann-Whitney U-test were used in SYSTAT software v10.2 (SYSTAT Software, Chicago, USA) for analysis of statistical significance between categorical variables and quantitative valuables, respectively. All tests were two-sided and the level of significance was set at *P*<0.05.

### Accession Numbers

Nucleotide sequences have been registered as #AB751399 to AB751499 in the DNA databank of Japan.

## Results

### CRF02_AG is the Predominant HIV-1 Strain in Koforidua, Ghana

**Figure 1:**
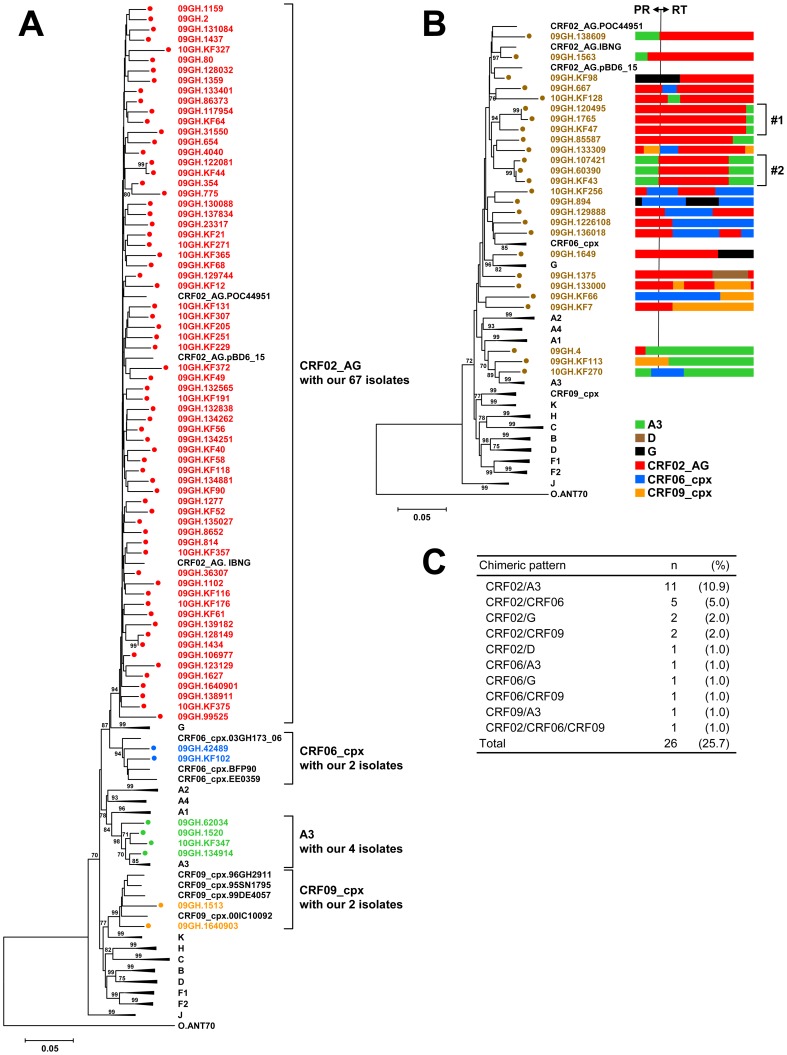
Molecular epidemiology of HIV-1 infections in Koforidua, Ghana. HIV-1 subtypes of 101 isolates were determined through the construction of phylogenetic trees, similarity plotting, and boot-scanning analyses. (A) Phylogenetic tree containing our 75 isolates classified into known subtypes and CRFs. (B) Phylogenetic tree containing our 26 URF isolates identified with unknown mosaic patterns of the *pol* gene. Two clusters of URF isolates are represented by #1 and #2. (C) Summary on the chimeric patterns of 26 URF isolates. The trees were constructed by the neighbor-joining method. Bootstrap values were calculated from 1,000 analyses, and values greater than 70% are shown at tree nodes. Our isolates are represented by colored circles, and subtype reference isolates are represented by their subtype and name. Scale bar represents nucleotide substitutions per site. HIV-1 group O isolate, ANT70, was used as the outgroup. CRF, circulating recombinant form; PR, protease; RT, reverse transcriptase; and URF, unique recombinant form.

During the study period, 101 HIV-1-infected patients were enrolled in this study. As shown in Table 2, 90 cases were adults (≧15 years old), including 59 newly diagnosed ART-naïve cases and 31 ART-experienced cases. The remaining 11 cases were children (<15 years old), among which were 10 newly diagnosed ART-naïve cases while one child was ART-experienced (Table 3). To understand the molecular epidemiology of HIV-1 infections in Ghana, we analyzed the *pol* gene sequences in detail through the construction of phylogenetic trees, similarity plotting, and boot-scanning analyses. Among the 101 cases, 75 (74.3%) were identified as HIV-1 subtypes and CRFs (Fig. 1A); 67 were CRF02_AG (66.3%), 4 were sub-subtype A3 (4.0%), 2 were CRF06_cpx (2.0%), and 2 were CRF09_cpx (2.0%). Thus, our analyses clearly showed the predominance of HIV-1 CRF02_AG in Koforidua.

**Table 2:** Demographic and clinical characteristics of ART-experienced and -naïve HIV-1-infected patients ≧15 years old (*n* = 90).

Characteristic		ART-experienced, *n* = 31	ART-naïve, *n* = 59	*P*
Age, years	Median (IQR)	38 (35–43)	42 (32–49)	0.251
Sex (%)	Female	18 (58.1)	38 (64.4)	0.649
	Male	13 (41.9)	21 (35.6)	
Risk factor for HIV infection (%)	Heterosexual contact	31 (100.0)	57 (96.6)	0.543
	Transfusion	0 (0.0)	2 (3.4)	
HIV serology (%)^a^	HIV-1 positive	29 (93.5)	59 (100.0)	0.116
	HIV-1 and -2 positive	2 (6.5)	0 (0.0)	
CD4^+^ T-cell count, cells/µl	Median (IQR)	230 (87–376)	237 (86–426)	0.750
HIV-1 viral load, log_10_ copies/ml	Median (IQR)	3.7 (3.3–4.1)	4.4 (3.7–5.0)	0.006
HIV-1 genotype (%)	CFR02_AG	18 (58.1)	42 (71.2)	0.638
	A3	2 (6.5)	2 (3.4)	
	CRF06_cpx	1 (3.2)	1 (1.7)	
	CRF09_cpx	1 (3.2)	1 (1.7)	
	URF	9 (29.0)	13 (22.0)	
ART regimen (%)				−
First line	d4T+3TC+EFV	10 (32.3)	−	
	AZT+3TC+NVP	10 (32.3)	−	
	AZT+3TC+EFV	7 (22.6)	−	
	d4T+3TC+NVP	3 (9.7)	−	
Second line	AZT+3TC+NFV	1 (3.2)	−	
Duration of ART, months	Median (IQR)	16.1 (6.8–30.3)	−	−
Adherence (%)^b^	Good	17 (54.8)	−	−
	Satisfactory	8 (25.8)	−	
	Poor	6 (19.4)	−	

ART, antiretroviral therapy; AZT, zidovudine; CRF, circulating recombinant form; d4T, stavudine; EFV, efavirenz; IQR, interquartile range; NFV, nelfinavir; NVP, nevirapine; 3TC, lamivudine; and URF, unique recombinant form.

a HIV serology was determined using New LAV Blot I and II (Bio-Rad Laboratories, Marnes-la-Coquette, France).

b Good, 100% pills taken; Satisfactory, ≧95%, but <100% pills taken; Poor, <95% pills taken.

**Table 3:** Demographic and clinical characteristics of HIV-1-infected patients <15 years old (*n* = 11)^a^.

Characteristic		Value
Age, years	Median (IQR)	5.0 (1.5–8.0)
Sex (%)	Female	6 (54.5)
	Male	5 (45.5)
CD4^+^ T-cell count, cells/µl	Median (IQR)	747 (474–1152)
HIV-1 viral load, log_10_copies/ml	Median (IQR)	4.3 (3.4–4.8)
HIV-1 genotype (%)	CRF02_AG	7 (63.6)
	URF	4 (36.4)
ART (%)	Naïve	10 (90.9)
	d4T+3TC+EFV^b^	1 (9.1)

ART, antiretroviral therapy; CRF, circulating recombinant form; d4T, stavudine; EFV, efavirenz; IQR, interquartile range; 3TC, lamivudine; and URF, unique recombinant form.

a All were HIV-1 seropositive alone, and their risk factor for infection was mother-to-child transmission.

b Only one case had been on treatment for 9.6 months.

Interestingly, the remaining 26 cases (25.7%) were identified as HIV-1 URFs (Fig. 1B). The most prevalent chimeric pattern was CRF02/A3 (*n* = 11, 10.9%), followed by CRF02/CRF06 (*n* = 5, 5.0%), CRF02/G (*n* = 2, 2.0%), CRF02/CRF09 (*n* = 2, 2.0%), and 6 other patterns (Fig. 1C). Of note, two interesting clusters were found in the phylogenetic tree of URF isolates (Fig. 1B). Cluster #1 with three isolates, 09GH.120495, 09GH.1765, and 09GH.KF47, shared the same mosaic *pol* gene comprising a large PR and RT fragment of CRF02 and a short RT fragment of A3 (cluster #1, Fig. 1B). Cluster #2 with the other three isolates, 09GH.107421, 09GH.60390, and 09GH.KF43, shared the same mosaic *pol* gene comprising the PR fragment of A3 and two RT fragments of CRF02 and A3 (cluster #2, Fig. 1B). Our data suggest that the two URF clusters are candidates for a new CRF spreading in this area of Ghana.

### HIV-1 Drug-resistance Mutations are Highly Frequent among ART-experienced Cases with Virological Failure Status

Demographic and clinical characteristics of 31 ART-experienced adult cases are shown in Table 2. All patients except one (96.8%, 30/31) were treated with the first-line ART regimen of 2 NRTIs+NNRTI, and the remaining one (3.2%) with the second-line ART regimen of 2 NRTIs+PI. Their median duration of ART was 16.1 months (IQR, 6.8–30.3 months), and most cases maintained their adherence at a “good” or “satisfactory” level (80.6%, 25/31).

Among these ART-experienced adult cases, 22 cases (71.0%) possessed one or more HIV-1 drug-resistance mutations (Table 4). The most prevalent drug-resistance pattern was 2-class resistance to NRTI and NNRTI (*n* = 13, 41.9%), followed by 1-class resistance to NNRTI (*n* = 8, 25.8%). Of note, 3-class resistance was identified in one case (3.2%) treated with the second-line regimen AZT+3TC+NFV. This case possessed HIV-1 RT mutations M41L, V90I, A98G, M184V and T215Y, and the major NFV-resistance mutation L90M in PR. As shown in Table 4, the most prevalent drug-resistance mutation among the 31 cases was M184V (*n* = 12, 38.7%), followed by K103N (*n* = 9, 29.0%), and T215Y/F (*n* = 6, 19.4%). No drug-resistance mutation was detected in the remaining 9 cases (29.0%, Table 4), suggesting that acquisitions of drug resistance was not the primary cause of their virological failure. The cases with and without resistance did not differ significantly in their demographic characteristics.

**Table 4:** Frequency of HIV-1 drug-resistance mutations in ART-experienced and -naïve adult patients (≧15 years old) (*n* = 90)^a^.

Mutation	ART-experienced, *n* = 31 (%)	ART-naïve, *n* = 59 (%)
Any	22 (71.0)	6 (10.2)
NNRTI resistance	8 (25.8)	6 (10.2)
NRTI and NNRTI resistance	13 (41.9)	0 (0.0)
NRTI, NNRTI, and PI resistance	1 (3.2)	0 (0.0)
Transmitted drug resistance	Not applicable	0 (0.0)
None	9 (29.0)	53 (89.8)
NRTI-resistance mutation	14 (45.2)	0 (0.0)
A62V	1 (3.2)	0 (0.0)
M184V	12 (38.7)	0 (0.0)
TAMs	8 (25.8)	0 (0.0)
M41L	4 (12.9)	0 (0.0)
D67N	3 (9.7)	0 (0.0)
K70R	4 (12.9)	0 (0.0)
L210W	1 (3.2)	0 (0.0)
T215Y	5 (16.1)	0 (0.0)
T215F	1 (3.2)	0 (0.0)
K219Q	1 (3.2)	0 (0.0)
K219E	1 (3.2)	0 (0.0)
NNTRI-resistance mutation	22 (71.0)	6 (10.2)
V90I	4 (12.9)	4 (6.8)
A98G	5 (16.1)	0 (0.0)
K103N	9 (29.0)	0 (0.0)
V106I	0 (0.0)	1 (1.7)
V106A	1 (3.2)	0 (0.0)
V108I	1 (3.2)	0 (0.0)
E138A	1 (3.2)	2 (3.4)
Y181C	3 (9.7)	0 (0.0)
Y188L	2 (6.5)	0 (0.0)
G190A	2 (6.5)	0 (0.0)
P225H	3 (9.7)	0 (0.0)
M230L	1 (3.2)	0 (0.0)
PI-resistance major mutation	1 (3.2)	0 (0.0)
L90M	1 (3.2)	0 (0.0)

ART, antiretroviral therapy; NNRTI, non-nucleoside reverse-transcriptase inhibitor; NRTI, nucleoside reverse-transcriptase inhibitor; PI, protease inhibitor; and TAMs, thymidine analog-associated mutations.

a HIV-1 drug-resistance mutations were detected according to the latest definition of the International AIDS Society-USA panel [10]. For ART-naïve patients, transmitted drug resistance was defined according to the latest definition of the WHO drug-resistance surveillance [11].

**Figure 2:**
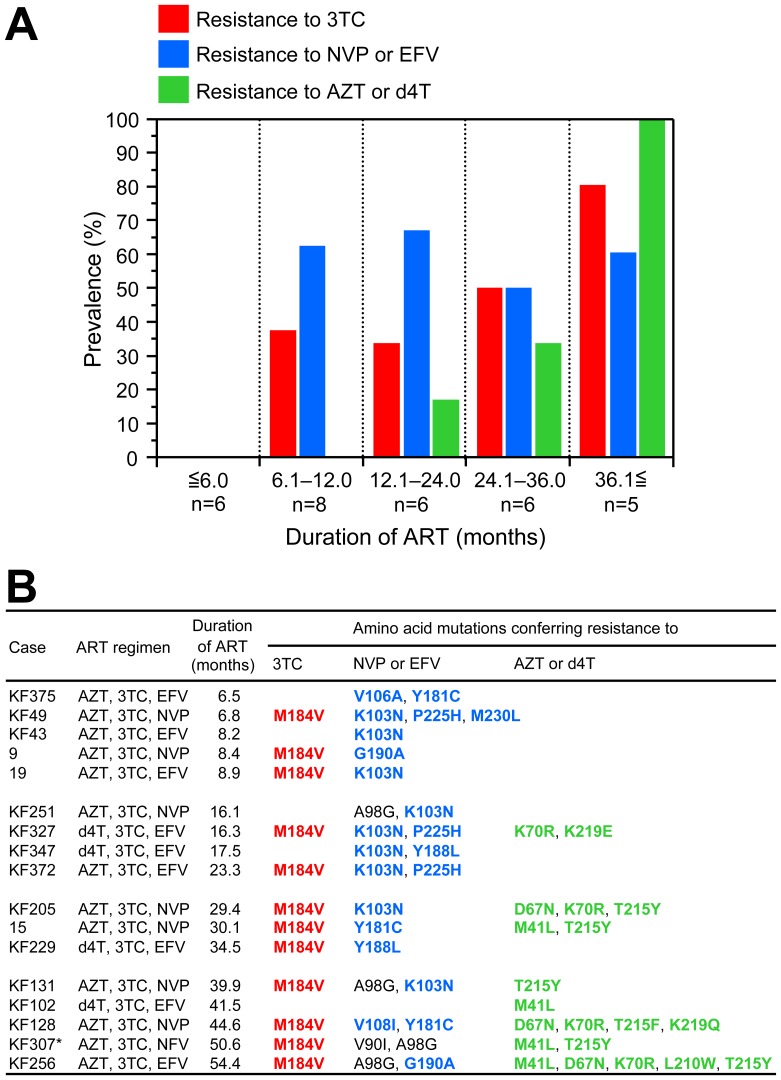
Prevalence of 3TC-, NVP-, EFV-, AZT-, and d4T-resistance mutations by duration of ART in 31 HIV-1-infected patients ≧15 years old. (A) Bar graph and (B) details of 17 patients identified with 3TC-, NVP-, EFV-, AZT-, and d4T-resistance mutations. HIV-1 drug-resistance mutations were detected according to the latest definition of the International AIDS Society-USA panel [10]. Amino acid mutations responsible for drug resistance are shown in bold and color coded with bar graph in A. *Major NFV-resistance mutation L90M was found in the protease in the case of KF307. ART, antiretroviral therapy; AZT, zidovudine; d4T, stavudine; EFV, efavirenz; NFV, nelfinavir; NVP, nevirapine; and 3TC, lamivudine.

Furthermore, we analyzed the chronological order of acquiring drug resistance to 3TC, NVP, EFV, AZT, and d4T. As shown in Fig. 2A, no mutation was found in any patients, even with viremia, who had received ART for ≦6.0 months (0%, 0/6). However, M184V mutation was detected in 37.5% (3/8) of patients with 6.1–12.0 months of ART, and the prevalence increased to 80.0% (4/5) at ≧36.1 months of ART (red bars in Fig. 2A and 2B). In the case of NVP and EFV resistance, K103N, V106A, V108I, Y181C/L, G190A, P225H, and M230L mutations were detected in more than half of patients after 6.0 months of ART (blue bars in Fig. 2A and 2B). Importantly, the prevalence and accumulation of thymidine analog-associated mutations (TAMs) appeared to be higher with longer duration of ART; 16.7% (1/6) at 12.1–24.0 months to 100% (5/5) at ≧36.1 months of ART (green bars in Fig. 2A and 2B).

### Low HIV-1 Drug-resistance Transmission in ART-naïve Cases

The general demographics of the 59 adult ART-naïve cases were similar to those of the treated cases, however pVL was significantly higher in the naïve cases (*P* = 0.006) (Table 2). Among the ART-naïve cases, no transmitted HIV-1 drug-resistance mutation was found (Table 4). However, polymorphisms at NNRTI-resistance mutation loci, V90I, E138A, and V106I, were found in 6 cases (10.2% in Table 4). Our data indicated that drug-resistant HIV-1 transmission events are still low in Koforidua, Ghana.

Eleven children infected with HIV-1 through mother-to-child transmission were also analyzed in our study (Table 3). Their median age was 5.0 years (IQR, 1.5–8.0 years), and 10 of these cases were ART naïve. The remaining case had been treated with d4T+3TC+EFV for 9.6 months but had become viremic. In this case, both 3TC-resistance (M184V) and EFV-resistance (V108I and G190S) mutations were detected (Table 5). Importantly, among the 10 ART-naïve children, a 1.5-year-old case had K103N and G190S NNRTI-resistance mutations (Table 5), suggesting the importance of HIV-1 drug-resistance testing in infants.

**Table 5:** HIV-1 drug-resistance mutations in patients <15 years old (*n* = 11)^a^.

ART	*n*	Amino acid mutations associated with	
		NNRTI resistance	NRTI resistance
Naïve	5	−	−
	2	V90I, V106I	−
	2	V90I	−
	1	**K103N**, **G190S**	−
d4T+3TC+EFV	1	K101E, V106I, V108I, G190S	M184V

ART, antiretroviral therapy; d4T, stavudine; EFV, efavirenz; NNRTI, non-nucleoside reverse-transcriptase inhibitor; NRTI, nucleoside reverse-transcriptase inhibitor; and 3TC, lamivudine.

a HIV-1 drug-resistance mutations were detected according to the latest definition of the International AIDS Society-USA panel [10]. For ART-naïve patients, transmitted drug-resistance (shown in bold and underlined) was detected according to the latest definition of the WHO drug-resistance surveillance [11].

## Discussion

Our results present a profile of the circulating subtypes and prevalence of drug resistance for HIV-1 infections in Koforidua, Ghana. The data clearly demonstrate the predominance of HIV-1 CRF02_AG (66.3%, *n* = 67) in the region (Fig. 1A). Our results, combined with three previous reports on the domination of CRF02_AG in Ghana between 1994 and 2004 [5], [6], [16], indicate that CRF02_AG has stabilized and maintained its predominance in the region for nearly 12 years. However, our study identified 26 isolates (25.7%) as URFs (Fig. 1B), indicating that active viral recombinations are ongoing in Ghana. Interestingly, a similar prevalence (25.1%) of HIV-1 URFs was reported from other cities in Ghana, Accra, Agomanya, and Atua [6]. Taken together, these data thus highlight the importance of HIV-1 URFs in understanding the dynamics of the HIV-1 epidemic in Ghana.

Regarding the situation of HIV-1 drug resistance in Ghana, most of the 31 patients on treatment with virological failure (*n* = 22, 71.0%) had HIV-1 drug-resistance mutations, suggesting that drug-resistant HIV-1 is the major risk factor for virological failure. Furthermore, nearly half of the cases (45.2%, 14/31) had both NRTI- and NNRTI-resistance mutations (Table 4), a pattern that is consistent with that observed in a recent systematic review on treatment-failure cases in sub-Saharan Africa [17], where M184V/I, K103N, and T215Y/F mutations predominate.

Regarding the timing of drug-resistance acquisition, our data demonstrated that 3TC-, NVP-, and EFV-resistance mutations were selected earlier (6.1–12.0 months) than AZT- and d4T-resistance mutations (12.1–24.0 months). Importantly, the prevalence of TAMs increased from 16.7% (1/6) at 12.1–24.0 months to 100% (5/5) at ≧36.1 months. As the accumulation of TAMs confers cross-resistance not only to the first-line NRTIs (AZT, d4T, and 3TC), but also to the second-line NRTIs (ABC, TDF, and ddI) to some extent [18], their accumulation should be avoided by conducting drug-resistance testing earlier and appropriately switching the regimen, once virological failure is suspected.

As no transmitted HIV-1 drug-resistance mutation was found among the 59 newly diagnosed treatment-naïve adult cases, the transmission of drug-resistant HIV-1 appeared to be a rare event in Koforidua, Ghana. Comparing our data with that from other African countries with a similar background, roll-out time of ART, and coverage rate of ART (26.6% in Ghana) [2], the low prevalence of transmitted HIV-1 drug resistance is not surprising and understandable. However, we cannot exclude the possibility of low levels of transmitted HIV-1 drug resistance in our 59 ART-naïve adult cases. The results of our study are limited by using direct sequencing, which may not have been sensitive enough to detect minority drug-resistant variants hidden among the wild-type strains. Indeed, several studies have reported 2- to 3-fold higher prevalence of drug-resistance transmission with ultra-deep sequencing than with direct sequencing [19], [20], which can detect 1% minority populations [21]. Furthermore, as ultra-deep sequencing can better detect the presence of dual or multiple infections of HIV-1 subtypes compared with direct sequencing [22], [23], using such new technology may identify subtypes of 26 URFs.

Finally, an eventual increase of transmitted drug-resistance cases is anticipated in Ghana as well. Thus, access to HIV-1 genotypic drug-resistance testing should ideally be expanded along with the scale-up of ART programs. In addition, vertical transmission of drug-resistant HIV-1 was found in one of 10 newly-diagnosed treatment-naïve children, suggesting that expanded access to HIV-1 genotypic drug-resistance testing is also needed for programs to prevent mother-to-child transmission in Ghana.
